# A huge posteromedial mediastinal cyst complicated with vertebral dislodgment

**DOI:** 10.1186/1477-7819-4-56

**Published:** 2006-08-22

**Authors:** Ilias A Kouerinis, George C Zografos, Dimitrios N Exarchos, Nikolaos T silimingas, Michalis E Argiriou, Jordan T Manoussaridis, Evangelos P Misiakos, Constantine I Fotiadis, Ion P Bellenis

**Affiliations:** 1 Department of Cardiothoracic Surgery, University of Thessaly, Greece; 2 Department of Cardiothoracic Surgery, Evangelismos Hospital, Athens, Greece; 3 First and Third Propaudeutic Surgical Departments, University of Athens, Greece

## Abstract

**Background:**

Mediastinal cysts compromise almost 20% of all mediastinal masses with bronchogenic subtype accounting for 60% of all cystic lesions. Although compression of adjoining soft tissues is usual, spinal complications and neurological symptoms are outmost rare and tend to characterize almost exclusively the neuroenteric cysts.

**Case presentation:**

A young patient with intermittent, dull pain in his back and free medical history presented in the orthopaedic department of our hospital. There, the initial clinical and radiologic evaluation revealed a mediastinal mass and the patient was referred to the thoracic surgery department for further exploration. The following computed tomography (CT) and magnetic resonance imaging (MRI) shown a huge mediastinal cyst compressing the T4-T6 vertebral bodies. The neurological symptoms of the patient were attributed to this specific pathology due to the complete agreement between the location of the cyst and the nervous rule area of the compressed thoracic vertebrae. Despite our strongly suggestions for surgery the patient denied any treatment.

**Conclusion:**

In controversy with the common faith that the spine plays the role of the natural barrier to the further expansion of cystic lesions, our case clearly indicates that, exceptionally, mediastinal cysts may cause severe vertebral complications. Therefore, early excision should be considered especially in young patients or where close follow up is uncertain.

## Background

It is well-established that approximately two thirds of mediastinal cysts are related to compression of adjoining structures and obstruction of the distal lung [1,2]. Nevertheless, when located in the posterior mediastinum the spine is the natural barrier who limits their uncompromised expansion and neurological symptoms are entirely related with the neuroenteric subtype [3].

In the present report we describe a rare case of a huge mediastinal cyst complicated with vertebral dislodgment and intermittent neurological symptoms.

## Case presentation

A 22 year-old non-smoker student was presented in the orthopaedic department of our hospital complaining about dull intermittent pain in his back. According to his history, he used to experience periodically similar, although shorter and milder, episodes for the last few years controlled by ordinary analgesics. He had neither previous incidence of trauma nor any prior orthopaedic diagnosis. Both physical findings and ordinary laboratory tests were unremarkable, but plain chest X-ray examination revealed an extensive, probably cystic, lesion located in the right posteromedial mediastinum. Focusing on this new finding the patient was referred to our department of thoracic surgery for further exploration.

**Figure 1:**
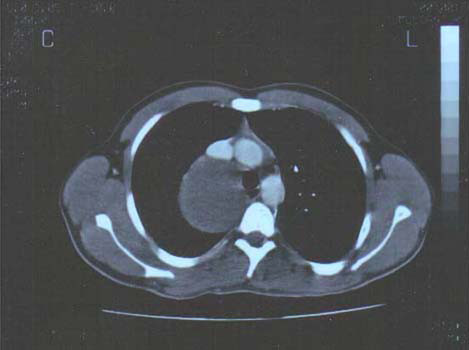
CT scan post contrast media injection reveals a huge low density compressive lesion in the posteromedial mediastinum. Notice the dislodgment of the thoracic vertebral body, the different dimensions of the spinal foramens and the different angles between the vertebral body and the traverse apophyses.

**Figure 2:**
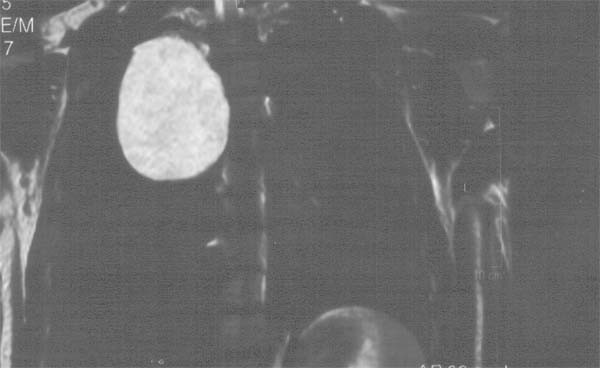
T2-W MRI scan set the diagnosis of the bronchogenic cyst and the vertebral dislodgment in the saggital section.

Our meticulous investigation of his medical history concerning any supplement general and respiratory symptoms proved negative. The patient had no evidence of weight loss, fever, persistent cough, haemoptysis, exposure to industrial agents or spore inhalation. The following computed tomography (CT) established the cystic nature of the mass (Hounsfield Units 38–40) which was also supported from the negative tumor markers. Surprisingly, the cyst was compressing not only the soft tissues-lung parenchyma, trachea and superior vena cava (SVC) – but also the thoracic vertebral bodies (Figure 1). These findings were also confirmed with magnetic resonance imaging (MRI) which established the diagnosis of vertebral dislodgment in the saggital section as well(Figure 2). The observed agreement between the area of the pain and the spinal deformity (T4-T6), indicated the cystic lesion as the leading cause of the symptoms, despite the lack of any previous radiographic data supporting so. Nevertheless, notwithstanding our strongly suggestions for surgery, the patient denied any treatment.

## Discussion

It is well known that the dimensions of cystic lesions may widely vary according to the dispensability of the adjacent tissues. On the other hand vertebral bodies seem to play the role of a natural barrier which tends to compromise their further expansion.

Controversial to this common experience the mediastinal cyst of our young patient became huge resulting not only soft tissues' but also vertebral column's compression. Accordingly, the described intermittent pain can be attributed to the narrowing of the contralateral vertebral foramens and the spinal dislodgment (Figure 1).

It is well established that spinal and neurological complications tend to characterize almost exclusively the neuroenteric cysts [3]. More specific neuroenteric cysts can be associated with cervical and upper thoracic vertebral anomalies such as scoliosis, anterior spina bifida, hemivertebrae, butterfly vertebrae or vertebral fusion or scoliosis [3,4]. The vertebral anomaly is commonly cephalad to the cyst as the esophagus descends during fetal development [5].

Nevertheless, the differential diagnosis between the variety of cystic masses of foregut origin-bronchogenic, esophageal and neuroenteric-based on radiographic criteria only can be proved difficult. Neuroenteric cysts rarely exhibit calcification and the absence of a meningocele, of cystic extension into the vertebral foramen or into the spinal canal may characterize a posterior mediastinal cyst as the paravetrtebral bronchogenic subtype [6]. On the other hand bronchogenic cysts may be calcified, while esophageal ones are found mostly along the lower third of thoracic esophagus with predilection for the right chest [4]. According to all above we would characterize our mediastinal cyst as neuroenteric or less probable as a bronchogenic.

Transtracheal needle biopsy (TTNB) and cytopathological examination of the collected fluid can establish the benign nature of a cyst and much more its' exact histology [4]. Nevertheless, this was not possible in our case due to complete lack of consent from the part of the patient.

Following the diagnosis of a mediastinal cyst, the treatment options are many. Different authors suggest different approaches: CT guided or mediastinoscopic drainage, video-assisted thoracic surgery (VATS) or open thoracotomy [7,8]. Nevertheless, and regardless of the scheduled treatment option, it has become evident that the management of mediastinal cysts should be prompt if patient's follow up is uncertain.

## Conclusion

Along with the possibility of malignant degeneration [9] our case clearly indicates that, if neglected, mediastinal cysts have the potency to become huge and appear unexpected complications [10,11]. Cervical and upper thoracic vertebral anomalies such as scoliosis, anterior spina bifida, hemivertebrae, butterfly vertebrae or vertebral fusion or scoliosis are outmost rare and tend to characterize the neuroenteric subtype [3,4]. The observed vertebral anomaly is commonly cephalad to the cyst as the esophagus descends during fetal development [5].

## Competing interests

The author(s) declare that they have no competing interests.

## Authors' contributions

**IAK **was the main surgeon managing the case. **GCZ, NT, MEA **were the assist surgeons managing the case. **DNE **performed the imaging and provided the CT pictures. **JTM **set the initial diagnosis, **EPM **helped in preparing the draft, **CIF **edited the final manuscript. **IPB **collection, analysis nd interpretation of data

All authors read and approved the manuscript.
